# The outcome of an attachment-based infant mental health therapeutic play programme on infant temperament, parent-infant relationship & maternal reflective functioning

**DOI:** 10.1192/bjo.2021.713

**Published:** 2021-06-18

**Authors:** Joy Malinit

**Affiliations:** Philippine Children's Medical Center, University of the East Ramon Magsaysay Memorial Medical Center

## Abstract

**Aims:**

In the Philippines, there is a need for preventive, early intervention programs for perinatal and infant mental health. This is the first local study that investigated an attachment-based, therapeutic play programme (Baby Bonding) on infant temperament, parent-infant relationship and maternal reflecting functioning.

**Background:**

This study was an effort towards bridging the “10/90 gap in infant mental health research” wherein 90% of the world's infants are born in low- middle-income countries (Population Reference Bureau, 2013b) and “only 10% of the worldwide spending on health research is directed towards the problems that primarily affect the poorest 90% of the world's population (Tomlinson et al., 2014).

**Method:**

Phase I involved local validation of the Parent-Rated Outcome Measures (PROM)- Infant Characteristics Questionnaire (ICQ), Mother Object Relations Scale (MORS) and Parental Reflective Functioning Questionnaire (PRFQ).Healthy mother-baby dyads, from the low socio-economic stratum, were screened using Parent Evaluation of Developmental Status (PEDS) and Hospital and Anxiety Depression Scale (HADS).

Phase II carried out randomized controlled design wherein mother-baby dyads were enrolled either in the usual care group or the 6-weekly Baby Bonding intervention.

**Result:**

102 mothers answered the PROM. Their responses constituted the training set of the study tools. Baseline responses of the mothers from the usual care (N = 51) and intervention (N = 53) groups formed the evaluation set for the Filipino- translated instruments. In both the training and evaluation sets, if certain identified questions were to be removed from the PROM, better and acceptable Cronbach values were consistently generated.

There were no statistical differences on parent-infant relationship and reflective functioning between the usual care and intervention group. There was movement of the intervention group from high-challenging onto intermediate to low-levels of challenging temperament in the dull-dimension of the ICQ after 6 sessions. In comparison, infants in the control group remained in the high-challenging temperament after 6 weeks of usual care.

**Conclusion:**

Linguistically validated study instruments (ICQ and MORS) provided accurate assessments of infant temperament and parent-child relationship. The Filipino-translated PRFQ has limited validity in evaluating parental reflective functioning (RF). “On-line” measures that video mother-baby interactions could have better captured changes in RF. As measured by the dull dimension of the ICQ, the Baby Bonding programme improved sociability of the infants (7 months or younger).

